# ADDRESSING THE COMPLEX DRIVING NEEDS OF AN AGING POPULATION: THE RISE OF AUTONOMOUS VEHICLES

**DOI:** 10.1093/geroni/igad104.3737

**Published:** 2023-12-21

**Authors:** David Carr, Hannes Devos, Abiodun Akinwuntan, Ganesh Babulal

**Affiliations:** Washington University in St. Louis, St. Louis, Missouri, United States; Kansas University Medical Center, Kansas City, Kansas, United States; KU School of Medicine, Kansas City, Kansas, United States; Washington University School of Medicine, St. Louis, Missouri, United States

## Abstract

Eighty-eight million Americans will be age 65 or older by 2050 and 25% of all drivers will be ≥ 65 years old. Technology has the potential to address some issues such as the inability to drive in advanced age. We postulate that continuing to drive with the use of Advanced Driver Assistance Systems (ADAS) and/or maintaining social connectedness with the use of autonomous vehicles (AVs) have the potential to slow down or reduce the impact of age-related diseases and enhance social mobility. ADAS are electronic technologies that assist drivers in safely operating a vehicle by detecting crash risk stimuli and reducing driver error. Older drivers’ willingness to use ADAS depends on individual perceived usefulness, safety, ease-of-learning, and anxiety. Based on the SAE levels of automation (0-5) , level 3-5 AVs can detect, evaluate, and respond to the vehicle environment without human involvement. Still, ADAS and AV technology cannot completely supplant all human driving and it cannot independently determine crash risk and safety. Comprehensive studies are needed to prove the effectiveness of ADAS in reducing crash risk among older drivers, especially those who are most vulnerable. In addition, AV manufacturers need to take into account the requirements and needs of providing this type of mobility service to older adults, especially those with chronic neurodegenerative diseases like dementia. If these technologies are eventually shown to be safe and embraced by older adults, they have the potential to delay driving cessation, prolong quality of life and maintain social connectedness.

